# Significado Prognóstico de Marcadores Associados à Nutrição na Insuficiência Cardíaca com Fração de Ejeção Preservada: Uma Revisão Sistemática e Metanálise

**DOI:** 10.36660/abc.20220523

**Published:** 2023-04-27

**Authors:** Ying Meng, Zhengyi Zhang, Tong Zhao, Dekui Zhang

**Affiliations:** 1 Departamento de Medicina Geral Segundo Hospital Universidade de Lanzhou Lanzhou Gansu China Departamento de Medicina Geral, Segundo Hospital da Universidade de Lanzhou, Lanzhou, Gansu – China; 2 Departamento de Ortopedia Primer Hospital Universidade de Lanzhou Lanzhou Gansu China Departamento de Ortopedia, Primer Hospital da Universidade de Lanzhou, Lanzhou, Gansu – China; 3 Departamento de Gastroenterologia Segundo Hospital Universidade de Lanzhou Lanzhou Gansu China Departamento de Gastroenterologia, Segundo Hospital da Universidade de Lanzhou, Lanzhou, Gansu – China

**Keywords:** Insuficiência Cardíaca, Prognóstico, Desnutrição, Volume Sistólico, Revisão Sistemática, Epidemiologia, Mortalidade

## Abstract

**Fundamento:**

O significado prognóstico dos indicadores nutricionais em pacientes com insuficiência cardíaca com fração de ejeção preservada (ICFEP) não é claro.

**Objetivo:**

Esta revisão sistemática e metanálise teve como objetivo avaliar o valor prognóstico da albumina sérica (AS), o índice de risco nutricional geriátrico (IRNG) e o índice nutricional prognóstico (INP) em pacientes com ICFEP.

**Método:**

Os bancos de dados PubMed, Embase, The Cochrane Library e Web of Science foram sistematicamente pesquisados para todos os estudos publicados até janeiro de 2022. O significado prognóstico de IRNG, GNRI e INP para ICFEP foi explorado. A taxa de risco agrupada (HR) e o intervalo de confiança (IC) de 95% foram estimados usando o software STATA 15.0. A Ferramenta de Estudos de Qualidade de Prognóstico foi usada para avaliar a qualidade dos estudos.

**Resultados:**

Nove estudos preencheram os critérios de inclusão e 5.603 adultos com ICFEP foram incluídos na metanálise. As análises mostraram que uma diminuição de AS ou IRNG estava significativamente relacionada à alta mortalidade por todas as causas (HR: 1,98; 95% IC: 1,282–3,057; p = 0,002; e HR: 1,812;95% IC: 1,064–3,086; p = 0,029, respectivamente). Além disso, uma AS mais baixa indica um resultado composto ruim de mortalidade por todas as causas e reinternação por IC (HR: 1,768; IC 95%: 1,483–2,108; p = 0,000), e um IRNG mais baixo foi significativamente associado a alta mortalidade cardiovascular (HR: 1,922; 95% IC: 1,504–2,457; p = 0,000). No entanto, um INP mais baixo não se correlacionou com mortalidade por todas as causas (HR: 1,176; IC 95%: 0,858–1,612, p=0,314).

**Conclusões:**

Nossa metanálise indica que AS e IRNG podem ser indicadores úteis para prever o prognóstico de pacientes com ICFEP.

## Introdução

Insuficiência cardíaca com fração de ejeção preservada (ICFEP) tornou-se uma forma cada vez mais comum de insuficiência cardíaca (IC). Estudos epidemiológicos mostraram que a proporção de ICFEP na população com IC aumentou de 41% em 1985-1994 para 56,17% em 2005-2014.^1^ Enquanto isso, estudos observacionais sugerem que a ICFEP está associada a alta morbidade e alta taxa de hospitalização.^2^ Esta condição tornou-se um fardo grave para a saúde pública, mas, infelizmente, não existem estratégias terapêuticas eficazes.

Pacientes com ICFEP geralmente são idosos com muitas complicações, incluindo hipertensão, diabetes, desnutrição,^3 - 5^ e problemas nutricionais relacionados a uma piora da IC. A desnutrição leva à inflamação sistêmica por meio de citocinas ativadas que podem estimular o sistema nervoso.^6 - 8^ Tudo isso está muito associado à progressão da IC. Uma variedade de indicadores pode ser usada para avaliar o risco nutricional. A albumina sérica (AS) é um indicador comum de avaliação nutricional, mas é suscetível a variações em doenças sistêmicas. O índice de risco nutricional geriátrico (IRNG) é usado para avaliar o estado nutricional com base no peso, altura e nível de AS,^9^ e o índice nutricional prognóstico (INP) é usado para avaliar o estado nutricional com base no nível de AS e na contagem de linfócitos.^10^ Esses índices multidimensionais são considerados mais precisos e abrangentes. Vários estudos têm mostrado o valor preditivo destes indicadores para vários resultados clínicos.^11 - 15^ A pesquisa sobre IC mostrou que esses índices também podem prever resultados em pacientes com insuficiência cardíaca com fração de ejeção reduzida (ICFEr).^16 - 19^ No entanto, o significado prognóstico dos indicadores nutricionais em pacientes com ICFEP não foi determinado, e os estudos que investigam o valor clínico da AS na predição do resultado da ICFEP têm resultados conflitantes.^20 , 21^ Não há revisões sistemáticas mostrando a relação entre os índices nutritivos e o prognóstico da ICFEP. Portanto, nossa revisão sistemática e metanálise foram projetadas para avaliar o valor prognóstico de AS, IRNG e INP em pacientes com ICFEP.

## Métodos

Esta revisão foi realizada seguindo as diretrizes de Itens de Relatório Preferidos para Revisões Sistemáticas e Metanálises.^22^ Foi registrada no Registro Prospectivo Internacional de Revisões Sistemáticas sob o ID de registro: CRD42021238546.

### Pesquisa de estudo

Os bancos de dados PubMed, Embase, The Cochrane Library e Web of Science foram sistematicamente pesquisados para todos os estudos sobre o significado prognóstico dos parâmetros nutricionais entre pacientes com ICFEP publicados até janeiro de 2022. Os seguintes termos de pesquisa foram usados (“albumina” OU “ALB” OU “hipoalbuminemia” OU “índice de risco nutricional geriátrico” OU “GNRI” OU “índice nutricional prognóstico” OU “PNI”) E (“insuficiência cardíaca com fração de ejeção preservada” OU “ HFpEF “ OU “ insuficiência cardíaca diastólica” OU “insuficiência cardíaca com fração de ejeção normal”). Além disso, examinamos as listas de referências de estudos selecionados e revisões sistemáticas relacionadas para identificar estudos relevantes.

### Critério de seleção

Dois autores (MY e ZT) realizaram o processo de seleção dos estudos de forma independente, e qualquer discordância foi discutida. Os critérios de inclusão foram os seguintes: 1. Pacientes adultos (>18 anos de idade) com ICFEP (a FE ventricular esquerda [FEVE] dos indivíduos com ICFEP incluídos neste estudo foi ≥ 40%); e 2. Estudos com informações prognósticas em um dos indicadores de avaliação nutricional (AS, IRNG ou INP). Os critérios de exclusão foram: 1. Pacientes com doença valvar cardíaca grave; 2. Pacientes com cardiopatias congênitas; 3. Pacientes com infarto agudo do miocárdio; 4. Pacientes com cor pulmonale; 5. Mulheres grávidas; 6. Dados incompletos mesmo após contato com os autores; e 7. Relatórios de caso e resumos de conferências.

### Extração de dados e avaliação de qualidade

Dois autores (MY e ZT) extraíram independentemente os seguintes dados dos estudos incluídos: o ano de publicação, primeiro autor, tamanho da amostra, desenho do estudo, duração do acompanhamento, idade média/mediana da população do estudo, fração de ejeção média, indicadores nutricionais, dados de desfecho, taxa de risco (HR) e intervalos de confiança de 95% (ICs) correspondentes.

A Ferramenta de Estudos de Qualidade de Prognóstico foi usada para avaliar o risco de viés,^15^ usando 6 parâmetros (participação no estudo, abandono do estudo, medição do fator prognóstico, medição do resultado, confundidor do estudo e análise e relatório estatístico), e os estudos foram classificados como alto, moderado ou baixo risco de viés.

### Análise estatística

A análise estatística foi realizada com o programa STATA versão 15.0 (Stata Corporation, College Station, TX, EUA). HR e IC 95% foram considerados em relação ao tamanho do efeito de cada estudo. Quando o HR não estava disponível, reconstruímos a estimativa do HR e sua variação a partir das curvas de sobrevivência de Kaplan-Meier pelo Engauge Digitizer. A heterogeneidade estatística foi avaliada usando o teste qui-quadrado Q e a estatística^23^ I^2^, onde I^2^ > 50% e p<0,05 indicaram heterogeneidade entre os estudos. Um modelo de efeitos fixos foi aplicado se não houvesse heterogeneidade significativa; caso contrário, um modelo de efeitos aleatórios foi usado. O teste de Egger avaliou o viés de publicação. Valores de p inferiores a 0,05 foram considerados estatisticamente significativos.

## Resultados

### Pesquisa e características do estudo

**Figura 1:**
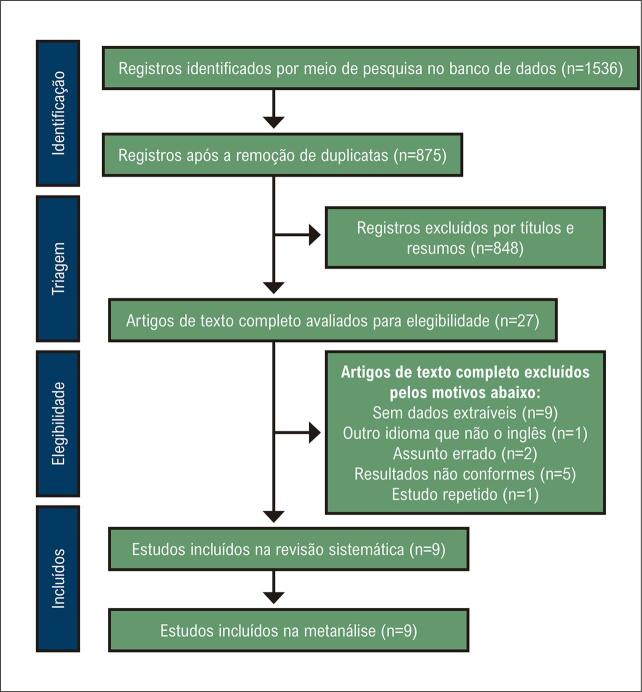
– Fluxograma de seleção de literatura.

A Figura 1 fornece uma seleção de pesquisa detalhada de estudos para esta metanálise. Identificamos 1.536 publicações por meio de uma pesquisa em banco de dados online; 661 foram excluídos devido à duplicação. Após a triagem dos títulos e resumos, foram excluídos 848 registros. O texto completo dos 27 estudos restantes foi revisado e avaliado em detalhes. Finalmente, incluímos 9 artigos nesta metanálise.^20 , 24 - 31^

As características dos estudos incluídos estão listadas na Tabela 1 . Dos nove estudos, cinco eram prospectivos e quatro eram retrospectivos. Todos os estudos foram publicados entre 2012 e 2020, seis foram conduzidos na Ásia e três na América do Norte. A análise incluiu 5.603 adultos que foram acompanhados por um ano a 5,8 anos em média. A idade média dos participantes variou de 32 a 98 anos. Esses estudos usaram vários pontos de corte de FEVE na população com ICFEP variando de 40% a 50%. Dois estudos usaram um limite de 40%, um usou 45% e seis usaram 50%. Três índices nutritivos foram usados nesses estudos selecionados; cinco estudos mediram a AS, quatro estudos mediram o IRNG e dois estudos mediram o INP para avaliar a desnutrição.

**Tabela 1:** – Características dos estudos incluídos na metanálise

	Liu	Vasiliki	Toshiyuki	Stuart	Yoshiharu	Isao	Masatoshi	Shih-Chieh	Yu-Lun
Ano	2012	2018	2018	2020	2013	2019	2019	2019	2017
País	China	EUA	Japão	EUA	Japão	Japão	EUA	China	China
Design de estudo	Prospectivo	Retrospectivo	Prospectivo	Prospectivo	Retrospectivo	Prospectivo	Retrospectivo	Retrospectivo	Prospectivo
Período de inclusão	Junho de 2006 a dezembro de 2009	Janeiro de 2012 a abril de 2012	Novembro de 2012 a março de 2015	-	Janeiro de 2004 a abril de 2011	Junho de 2012 a março de 2015	Agosto de 2006 a janeiro de 2012	Março de 2021 a dezembro de 2014	Outubro de 2003 a dezembro de 2012
Acompanhamento	12m	2 anos	731d	57,6 m	2,1 anos	503.5d	2,9 anos	1255d	31,5m
Número	576	445	535	118	152	110	1677	1120	870
Mulheres, %	64%	57,80%	50%	92%	46,10%	46,40%	50,80%	60,60%	-
Idade média	77±10	73（63,83)	80 (73-84)	65,42 ±9,49	77±11	78,5±7,2	72,12±2,49	77.2	-
Classe NYHA II-III, %	83%	24%	74%	-	83,40%	90,90%	-	86,20%	-
FEVE	≥50%	>40%	≥50%	>50%	≥40%	≥50%	≥45%	≥50%	≥50%
Índice nutricional	AS	AS	AS	AS	IRNG	IRNG	IRNG	AS, IRNG, INP	INP
Corte	34g/L	34g/L	-	35g/L	92	92	-	AS:35g/L; IRNG:92; INP:38	39.3
Desfecho, HR (95% IC)	ACM,3.18 (2.27–4.45)	ACM,1.67 (1.28–2.18) CEP,1.69(1.13-2.53)	CEP,2.27(1.59-3.23)	CEP,1.61 (1.29-2.06)	ACM,2.667(1.527-4.651); CM, 2.469(1.248-4.902)	ACM,3.202 (1.295–7.918)	ACM:1,79 (1,33–2,42); CM:2,06(1,40–3,03)	ACM: AS:1,49 (1,18–2,22), IRNG:1,02 (1,01–1,03), INP:1,03 (1,01–1,05); CM: IRNG:1,69 (1,19–2,44)	ACM,1,43(1,08-1,90)
Variáveis de ajuste	Idade, sexo masculino, níveis de CR, SBP, história de DCV, história de DM, níveis de BUN, níveis de Hb, uso de ACEIs/ARBs	idade e sexo	-	-	-	idade e sexo	Classe funcional NYHA, hipertensão, DM, hospitalização IC, MI, acidente vascular cerebral, AF, qualquer câncer, uso de ACEIs/ARBs, betabloqueadores, níveis de Hb, níveis séricos de sódio, níveis de bilirrubina, eGFR	Idade, sexo, IMC, pressão arterial sistólica, frequência cardíaca, IC prévia, hipertensão, doença cardiovascular, diabetes e fibrilação atrial	-

ACM: mortalidade por todas as causas; CM: mortalidade cardiovascular; CEP: Desfecho composto (mortalidade por todas as causas e reinternação por IC); y:ano; m: mês; d: dia.

### Resultado da metanálise

#### AS

**Figura 2:**
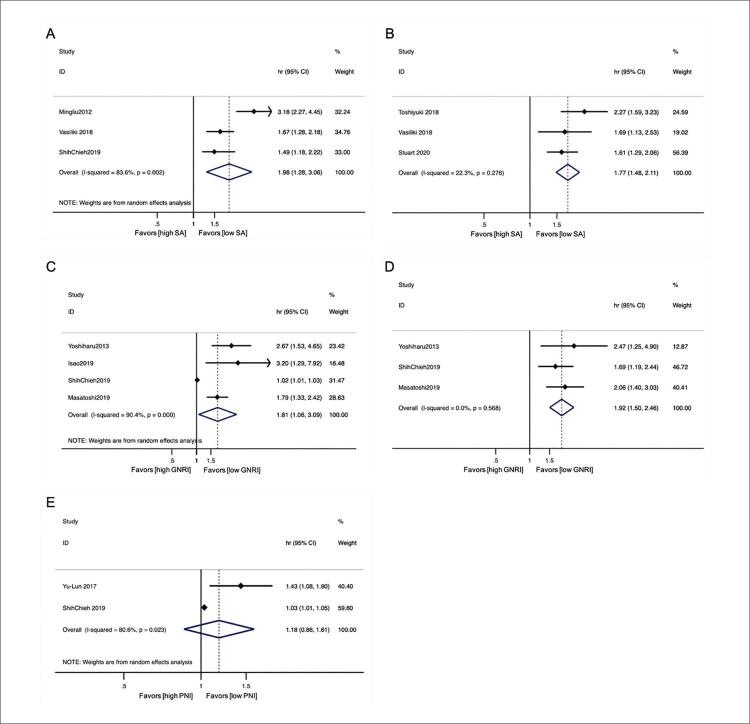
– A) Gráfico de Forrest da razão de risco (HR) para a associação entre albumina sérica (AS) e mortalidade por todas as causas; B) Gráfico de Forrest da taxa de risco para a associação entre AS e o desfecho composto de mortalidade por todas as causas e reinternação por IC; C) Gráfico de Forrest da taxa de risco para a associação entre índice de risco nutricional geriátrico (IRNG) e mortalidade por todas as causas; D) Gráfico de Forrest da razão de risco para a associação entre IRNG e mortalidade cardiovascular; E) Gráfico de Forrest da taxa de risco para a associação entre índice nutricional prognóstico (INP) e mortalidade por todas as causas. A heterogeneidade entre os estudos foi determinada usando estatísticas I2 em um nível de significância de p < 0,05.

IC: intervalo de confiança; HR: taxa de risco.

Três estudos analisaram a mortalidade por todas as causas com AS. Depois de combinar HR, menor AS previu maior mortalidade por todas as causas no modelo de efeitos aleatórios (HR = 1,98; 95% IC = 1,282–3,057, p = 0,002; I^2^ = 83,6%; Figura 2A ), e o teste Egger (p = 0,584) não identificou viés de publicação. Três estudos analisaram o desfecho composto de mortalidade por todas as causas e reinternação por IC com AS, um modelo de efeitos fixos (HR = 1,768; 95% IC = 1,483–2,108, p = 0,000; I2 = 22,3%; Figura 2B ) foi estatisticamente significativo, e o teste de Egger (p = 0,661) não mostrou viés de publicação.

#### IRNG

Quatro estudos analisaram a mortalidade por todas as causas com IRNG. Após a combinação de HR, o IRNG mais baixo e a pior mortalidade por todas as causas foram previstos. Uma vez que foi observada uma heterogeneidade significativa entre os estudos individuais (I^2^ = 90,4%, p < 0,01), um modelo de efeitos aleatórios foi usado para obter o efeito de estimativa agrupada. A metanálise revelou um aumento significativo da mortalidade por todas as causas (HR: 1,812; IC 95%: 1,064–3,086, p = 0,029; Figura 2C ) para pacientes com ICFEP com baixo IRGN. No entanto, pode haver viés de publicação conforme suportado pelo teste de Egger (p = 0,014). Isso foi testado posteriormente pela análise Trim and Fill, e o resultado do HR agrupado não mudou. O viés não teve efeito no resultado da avaliação.

A mortalidade cardiovascular foi analisada em três estudos com IRNG. Dados abrangentes mostraram que IRGNs mais baixos estavam relacionados a maior mortalidade cardiovascular, e o modelo de efeitos fixos (HR = 1,922; 95% IC = 1,504–2,457, p = 0,000; I^2^ = 0,00%; Figura 2D ) foi estatisticamente significativo, e o teste de Egger (p = 0,41) não mostrou viés de publicação.

#### INP

O INP foi estimado usando um modelo aleatório em dois estudos, e o HR agrupado não revelou diferença estatística na mortalidade por todas as causas entre os pacientes com alto e baixo nível de INP (HR: 1,176; 95% IC: 0,858–1,612, p= 0,314, I2 = 80,6%; Figura 2E ), o teste de Eggert (p < 0,05) mostrou certo viés de publicação, como visto em alguns estudos.

#### Qualidade do estudo

A qualidade desses estudos foi avaliada de acordo com a Ferramenta de Estudos de Qualidade de Prognóstico; sete estudos classificaram qualidade moderada e dois estudos classificaram alta qualidade. Quatro estudos não registraram ou controlaram fatores de confusão relacionados aos resultados da avaliação e três não forneceram informações sobre perdas de seguimento. Os detalhes são fornecidos na Tabela 2 .

**Tabela 2:** – Ferramenta de Qualidade dos Estudos de Prognóstico para avaliar a qualidade dos estudos selecionados

Estudo, ano	Participação no estudo	Abandono do estudo	Medição do fator prognóstico	Medição do resultado	Confundimento do estudo	Análise estatística e relatórios	Total
MingLiu, 2012	M	L	M	M	H	M	M
Yoshiharu, 2013	M	L	M	M	L	M	M
Yu-lu, 2017	M	M	M	M	L	M	M
Vasiliki, 2018	M	L	M	M	M	M	M
Toshiyuki, 2018	M	M	M	M	L	M	M
Isao, 2019	M	M	H	H	H	H	H
ShihChieh, 2019	M	M	H	H	H	H	H
Masatoshi, 2019	M	M	M	M	M	M	M
Stuart, 2020	M	M	H	H	L	M	M

L: baixa qualidade; M: qualidade média; H: alta qualidade.

## Discussão

A desnutrição pode resultar em deficiência energética, hipofunção imunológica e danos nos tecidos e órgãos.^32^ Em comparação com pacientes bem nutridos, os pacientes desnutridos têm internações hospitalares mais longas, maiores taxas de readmissão e mortalidade.^33^ Os desequilíbrios de anabolismo e catabolismo no desenvolvimento da IC podem também levar à desnutrição. Pesquisas sugerem que 50% dos pacientes com IC crônica desenvolveram algum grau de desnutrição.^25^ O desequilíbrio entre o suprimento de nutrientes e as necessidades de energia resulta em metabolismo de energia celular prejudicado e afeta os sistemas metabólicos de todo o corpo. O consumo significativo de energia corporal pode causar caquexia cardíaca; foi relatado que 15% dos pacientes com IC manifestavam caquexia.^25^ Ao mesmo tempo, a caquexia cardíaca é considerada fator de risco para mortalidade em pacientes com IC.^34^

Como a importância da desnutrição em pacientes com ICFEP ainda não foi totalmente avaliada, avaliamos o papel de diferentes indicadores nutricionais (AS, IRNG e INP)na predição do prognóstico da doença em pacientes com ICFEP. Descobrimos que pontuações mais baixas de AS e IRNG foram significativamente associadas a maior mortalidade por todas as causas, e AS mais baixa também está associada a resultados compostos aumentados de mortalidade por todas as causas e taxas de hospitalização por IC. No entanto, não houve correlação entre menor INP e mortalidade por todas as causas. Os achados sugerem que AS e IRNG podem ser indicadores úteis para avaliar o prognóstico em um paciente com ICFEP.

A AS é um indicador simples e objetivo de avaliação nutricional, podendo refletir melhor a massa muscular e o armazenamento proteico.^35 , 36^ Também é considerado um marcador inflamatório.^37^ A AS desempenha um papel importante em muitos processos fisiológicos, incluindo a manutenção de uma pressão coloidosmótica estável e da integridade microvascular, entregando substância no corpo como uma proteína transportadora, e eliminando radicais livres e atividades anticoagulantes.^38^ Segundo levantamento de Liu et al.,^20^ a deficiência de AS foi observada em 30% dos pacientes com IC crônica.^20^ A hipoproteinemia pode promover o desenvolvimento de IC por causar edema pulmonar e miocárdico, retenção hídrica, resistência diurética, estresse oxidativo e inflamação.^39^ Um estudo multicêntrico incluindo adultos sem IC mostrou o importante papel da AS no desenvolvimento de IC, em que a hipoalbuminemia basal está associada a um risco aumentado de desenvolver IC durante o período de acompanhamento de 10 anos.^40^ No entanto, estudos produziram resultados conflitantes sobre a capacidade do AS de prever o prognóstico de pacientes com ICFEP. Liu et al.,^20^ sugerem que a hipoalbuminemia foi significativamente relacionada ao aumento do risco de morte para pacientes com ICFEP.^20^ No entanto, Shanmugam et al. mostram que a hipoalbuminemia não teve relação óbvia com a mortalidade em 1 ano em pacientes com ICFEP.^21^ Nossa metanálise revela que a hipoalbuminemia foi significativamente associada a uma alta taxa de mortalidade por todas as causas e hospitalizações por IC em pacientes com ICFEP, que sustentam que AS é um forte preditor de resultado adverso em pacientes com ICFEP.

O GNRI foi proposto por Bouillanne et al.,^41^ seus parâmetros básicos são AS e índice de massa corporal (IMC), e foi inicialmente usado para avaliar o risco nutricional em idosos, mas também foi útil para prever desfechos clínicos em diferentes condições patológicas. No entanto, também foi encontrado para prever resultados clínicos em diferentes condições patológicas.^42 , 43^ Seoudy et al.,^44^ sugerem que, em comparação com indivíduos saudáveis, o nível de biomarcadores cardiovasculares aumentou acentuadamente e a prevalência de IC crônica foi maior em pacientes com baixo IRNG.^44^ Além disso, a pesquisa mostrou que o IRNG foi associado à sobrecarga de volume,^45^ maior morte cardiovascular e taxas mais altas de reinternação^46 , 47^ em pacientes com IC. Nossa metanálise também indica que o baixo IRNG se correlaciona com uma alta taxa de mortalidade cardiovascular em pacientes com ICFEP. Alguns pesquisadores acreditam que o IRNG representa o estado de fragilidade dos pacientes causado por vários estressores sob distúrbios de múltiplos sistemas.^48 , 49^ Estudos têm demonstrado que pacientes com ICFEP apresentam maior taxa de mortalidade quando apresentam baixo IMC e baixa reserva proteica,^35 , 50^ e este mau estado nutricional pode representar a progressão da ICFEP.

O INP é um índice de avaliação nutricional que pode representar a síntese de proteínas e a função imunológica do corpo.^51^ O estado nutricional pode afetar o metabolismo e a função das células imunes, e a desnutrição pode levar à imunossupressão e afetar o prognóstico dos pacientes.^52^ O INP foi originalmente usado para avaliar o risco perioperatório de pacientes de cirurgia gastrointestinal.^53^ No entanto, pesquisas recentes mostram que o INP é um marcador prognóstico eficaz em pacientes com vários tumores malignos,^54^ IC aguda,^25^ e embolia pulmonar.^55^ Na nossa análise, apenas dois estudos puderam ser usados para a análise combinada do impacto do baixo INP na mortalidade por todas as causas da ICFEP, e os resultados falharam em mostrar uma correlação entre INP e ICFEP. Essa falta de correlação pode ser devida à heterogeneidade clínica, pois os pontos de corte para INP não são uniformes. No entanto, devido ao pequeno número de estudos incluídos e à indisponibilidade de mais análises de subgrupos, estudos de alta qualidade são necessários para avaliar o valor preditivo do INP no prognóstico da ICFEP.

Como a ICFEP é uma doença com alta heterogeneidade e processos patológicos complicados causados por múltiplas comorbidades que podem afetar o desenvolvimento da ICFEP, um único índice nutricional pode não prever com precisão o resultado em todos os pacientes. A avaliação abrangente de vários indicadores nutricionais pode fornecer informações prognósticas mais completas e aumentaria a capacidade de prever e estratificar o risco de ICFEP. Ao mesmo tempo, essa identificação de risco pode levar a uma melhor tomada de decisão clínica para retardar a progressão da doença, e a formulação de planos de intervenção nutricional também pode ajudar a melhorar o resultado clínico desses pacientes. Foi demonstrado que os suplementos nutricionais são bons para pacientes com IC crônica,^56 , 57^ porém mais estudos clínicos são necessários para verificar se estão diretamente relacionados ao prognóstico de pacientes com ICFEP.

### Limitações

Existem algumas limitações em nosso estudo. Existem relativamente poucos estudos relacionados; portanto, não poderíamos incluir tantos estudos avaliáveis quanto possível. Em nossa metanálise, definimos ICFEP como uma FEVE≥40%, o que pode causar alguma diferença nos resultados. Em alguns estudos, o HR e os ICs de 95% foram estimados por curvas de sobrevida de Kaplan-Meier, o que pode levar a um erro potencial. Além disso, os estudos apresentam certa heterogeneidade, que pode estar associada à inconsistência do valor de corte e fatores de confusão ajustados no cálculo da FC nos estudos incluídos. Além disso, como as doenças sistêmicas podem afetar o estado nutricional, isso também aumentará a heterogeneidade do estudo. Devido ao número e qualidade limitados dos estudos, mais estudos são necessários para avaliar o papel dos indicadores nutricionais na predição do prognóstico da ICFEP.

## Conclusão

Como é resumido na Figura central , esta metanálise fornece evidências da correlação entre os índices nutricionais, AS e IRNG, e o prognóstico de pacientes com ICFEP, mostrando que pacientes com ICFEP com baixo AS têm maior risco de morte por todas as causas e um risco maior de eventos de desfecho composto de morte por todas as causas e reinternação, e pacientes com ICFEP com baixo IRNG têm maior risco de morte por todas as causas e morte cardiovascular. Esses resultados indicam o valor preditivo de AS e IRNG no prognóstico de pacientes com ICFEP e podem ser indicadores de referência úteis para a avaliação do prognóstico de ICFEP.

**Figura Central:**
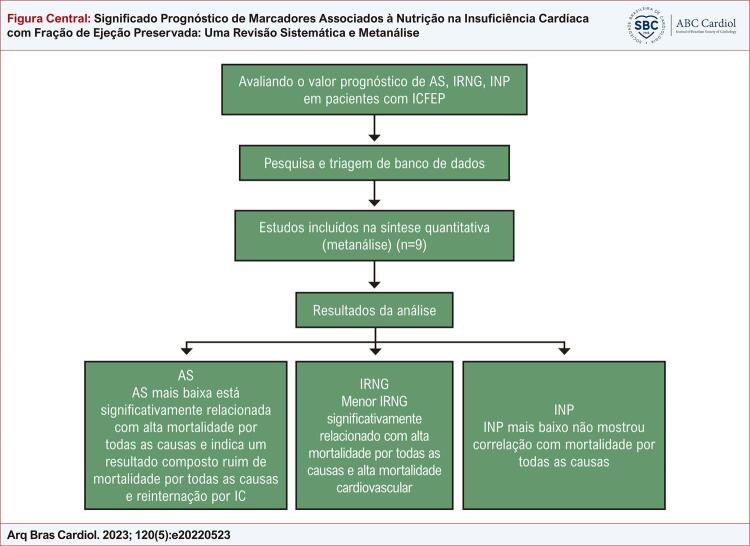
: Significado Prognóstico de Marcadores Associados à Nutrição na Insuficiência Cardíaca com Fração de Ejeção Preservada: Uma Revisão Sistemática e Metanálise
