# Supplementary material for: Significado Prognóstico de Marcadores Associados à Nutrição na Insuficiência Cardíaca com Fração de Ejeção Preservada: Uma Revisão Sistemática e Metanálise
Source: Arq Bras Cardiol. 2023 Apr 27;120(5):e20220523. [Article in Portuguese] doi: 10.36660/abc.20220523 (PMC10263396; doi:10.36660/abc.20220523)
Supplement: Translation

# Translation: Prognostic Significance of Nutrition-Associated Markers in Heart Failure with Preserved Ejection Fraction: A Systematic Review and Meta-Analysis

**Authors:** Ying Meng, Zhengyi Zhang, Tong Zhao, Dekui Zhang

**Affiliations:** 1 China Departamento de Medicina Geral, Segundo Hospital da Universidade de Lanzhou, Lanzhou, Gansu – China; 2 China Departamento de Ortopedia, Primer Hospital da Universidade de Lanzhou,Lanzhou, Gansu – China; 3 China Departamento de Gastroenterologia, Segundo Hospital da Universidade de Lanzhou, Lanzhou, Gansu – China

**Keywords:** Heart Failure, Prognosis, Malnutrition, Strpke Volume, Systematic Reviews, Epidemiology, Mortality

## Abstract

**Background:**

The prognostic significance of nutrition indicators in patients with heart failure with preserved ejection fraction (HFpEF) is unclear.

**Objectives:**

This systematic review and meta-analysis aimed to assess the prognostic value of serum albumin (SA), the geriatric nutritional risk index (GNRI), and the prognostic nutritional index (PNI) in patients with HFpEF.

**Methods:**

Databases of PubMed, Embase, The Cochrane Library, and Web of Science were systematically searched for all studies published up to January 2022. The prognostic significance of SA, GNRI, and PNI for HFpEF was explored. Pooled hazard ratio (HR) and 95% confidence interval (CI) were estimated using the STATA 15.0 software. The Quality of Prognosis Studies tool was used to assess the quality of studies.

**Results:**

Nine studies met the inclusion criteria, and 5603 adults with HFpEF were included in the meta-analysis. The analyses showed that a decreased SA or GNRI was significantly related to high all-cause mortality (HR: 1.98; 95% CI: 1.282–3.057; p = 0.002; and HR: 1.812;95% CI: 1.064–3.086; p = 0.029, respectively). Furthermore, a lower SA indicates a bad composite outcome of all-cause mortality and HF rehospitalization (HR: 1.768; 95% CI: 1.483–2.108; p = 0.000), and a lower GNRI was significantly associated with high cardiovascular mortality (HR: 1.922; 95% CI: 1.504–2.457;p = 0.000). However, a lower PNI did not correlate with all-cause mortality (HR: 1.176; 95% CI: 0.858–1.612, p=0.314).

**Conclusions:**

Our meta-analysis indicates that SA and GNRI may be useful indicators to predict the prognosis of patients with HFpEF.

## Introduction

Heart failure with preserved ejection fraction (HFpEF) has become an increasingly common form of heart failure (HF). Epidemiological studies have shown that the proportion of HFpEF in the HF population has increased from 41% in 1985-1994 to 56.17% in 2005-2014.^1^ Meanwhile, observational studies suggest that HFpEF is associated with high morbidity and rate of hospitalization.^2^ This condition has become a severe public health burden, but unfortunately, no effective therapeutic strategies exist.

Patients with HFpEF are usually elderly with many complications, including hypertension, diabetes, malnutrition,^3 - 5^ and nutritional problems related to a worse HF. Malnutrition leads to systemic inflammation via activated cytokines that can stimulate the nervous system.^6 - 8^ All these are greatly associated with the progression of HF. A variety of indicators can be used to assess nutritional risk. Serum albumin (SA) is a common indicator of nutritional assessment but is susceptible to variations in systemic diseases. Geriatric nutritional risk index (GNRI) is used to assess the nutritional status based on the weight, height, and level of SA,^9^ and prognostic nutritional index (PNI) is used to assess the nutritional status based on SA level and the lymphocyte count.^10^ These multidimensional indices are considered more accurate and comprehensive. Several studies have shown the predictive value of these indicators for various clinical outcomes.^11 - 15^ Research on HF has shown that these indices can also predict outcomes in patients with heart failure with reduced ejection fraction (HFrEF).^16 - 19^ However, the prognostic significance of nutritional indicators in patients with HFpEF has not been determined, and studies investigating the clinical value of SA in predicting the outcome of HFpEF have conflicting results.^20 , 21^ There are no systematic reviews showing the relationship between nutritive indexes and the prognosis of HFpEF. Therefore, our systematic review and meta-analysis were designed to evaluate the prognostic value of SA, GNRI, and PNI in patients with HFpEF.

## Methods

This review followed the Preferred Reporting Items for Systematic Reviews and Meta-Analyses guidelines.^22^ It was registered in the International Prospective Register of Systematic Reviews under the registration ID: CRD42021238546.

### Study search

PubMed, Embase, The Cochrane Library, and Web of Science databases were systematically searched for all studies about the prognostic significance of nutritional parameters among patients with HFpEF published till January 2022. The following search terms were used (“albumin” OR “ALB” OR “hypoalbuminemia” OR “geriatric nutritional risk index” OR “GNRI” OR “prognostic nutritional index” OR “PNI”) AND (“heart failure with preserved ejection fraction” OR “HFpEF” OR “diastolic heart failure” OR “heart failure with normal ejection fraction”). We additionally screened the reference lists of selected studies and related systematic reviews to identify relevant studies.

### Selection criteria

Two authors (MY and ZT) independently performed the study selection process, and any disagreement was discussed. The inclusion criteria were as follows: 1. Adult patients (>18 years old) with HFpEF (the left ventricular EF [LVEF] of HFpEF subjects included in this study was ≥ 40%); and 2. Studies with prognostic information on one of the nutritional assessment indicators (SA, GNRI, or PNI). The exclusion criteria were: 1. Patients with severe heart valve disease; 2. Patients with congenital heart diseases; 3. Patients with acute myocardial infarction; 4. Patients with cor pulmonale; 5. Pregnant women; 6. Incomplete data even after contacting the authors; and 7. Case reports and conference abstracts.

### Data extraction and quality assessment

Two authors (MY and ZT) independently extracted the following data from the included studies: the year of publication, first author, sample size, study design, follow-up duration, mean/median age of the study population, mean ejection fraction, nutritional indicators, endpoint data, hazard ratio (HR), and corresponding 95% confidence intervals (CIs).

The Quality of Prognosis Studies Tool was used to assess the risk of bias,^15^ using 6 parameters (study participation, study attrition, prognostic factor measurement, outcome measurement, study confounding, and statistical analysis and reporting), and the studies were rated as high, moderate, or low risk of bias.

### Statistical analyses

We performed the statistical analysis using STATA version 15.0 (Stata Corporation, College Station, TX, USA). HR and 95% CI were considered concerning the effect size of each study. When the HR was unavailable, we reconstructed the HR estimate and its variance from the Kaplan-Meier survival curves by Engauge Digitizer. Statistical heterogeneity was evaluated using the chi-squared Q test and I^2^statistic,^23^ where I^2^> 50% and p<0.05 indicated heterogeneity between studies. A fixed-effects model was applied if there was no significant heterogeneity; otherwise, a random-effects model was used. Egger’s test evaluated publication bias. P values of less than 0.05 were considered statistically significant.

## Results

### Study search and characteristics

Figure 1 provides a detailed search selection of studies for this meta-analysis. We identified 1536 publications through an online database search; 661 were excluded due to duplication. After screening the titles and abstracts, we excluded 848 records. The full text of the remaining 27 studies was reviewed and evaluated in detail. Finally, we included 9 articles in this meta-analysis.^20 , 24 - 31^

**Figure 1:** – Flow chart of literature selection.

The characteristics of the studies included are listed in Table 1 . Of the nine studies, five were prospective, and four were retrospective. All studies were published between 2012 and 2020, six were conducted in Asia, and three were in North America. The analysis included 5603 adults who were followed up for one year to 5.8 years on average. The subjects’ average ages ranged from 32 to 98 years. These studies used various LVEF cut-offs in the HFpEF population ranging from 40% to 50%. Two studies used a threshold of 40%, one used 45%, and six used 50%. Three nutritive indexes were used in these selected studies; five studies measured the SA, four studies measured the GNRI, and two studies measured the PNI to assess malnutrition.

**Table 1:** – Characteristics of studies included in the meta-analysis

	Liu	Vasiliki	Toshiyuki	Stuart	Yoshiharu	Isao	Masatoshi	Shih-Chieh	Yu-Lun
Year	2012	2018	2018	2020	2013	2019	2019	2019	2017
Country	China	USA	Japan	USA	Japan	Japan	USA	China	China
Study design	Prospective	Retrospective	Prospective	Prospective	Retrospective	Prospective	Retrospective	Retrospective	Prospective
Inclusion period	June 2006-December 2009	January 2012-April 2012	November 2012-March 2015	-	January 2004-April 2011	June 2012-March 2015	August 2006-January 2012	March 2021-December 2014	October 2003-December 2012
Follow up	12m	2y	731d	57.6m	2.1y	503.5d	2.9y	1255d	31.5m
Number	576	445	535	118	152	110	1677	1120	870
Women, %	64%	57.80%	50%	92%	46.10%	46.40%	50.80%	60.60%	-
Mean age	77±10	73(63,83)	80(73-84)	65.42 ±9.49	77±11	78.5±7.2	72.12±2.49	77.2	-
NYHA class II-III, %	83%	24%	74%	-	83.40%	90.90%	-	86.20%	-
LEVF	≥50%	>40%	≥50%	>50%	≥40%	≥50%	≥45%	≥50%	≥50%
Nutritional index	SA	SA	SA	SA	GNRI	GNRI	GNRI	SA, GNRI, PNI	PNI
Cut off	34g/L	34g/L	-	35g/L	92	92	-	SA:35g/L; GNRI:92; PNI:38	39.3
Outcome, HR(95% CI)	ACM,3.18 (2.27–4.45)	ACM,1.67 (1.28–2.18) CEP,1.69(1.13-2.53)	CEP,2.27(1.59-3.23)	CEP,1.61 (1.29-2.06)	ACM,2.667(1.527-4.651); CM, 2.469(1.248-4.902)	ACM,3.202 (1.295–7.918)	ACM:1.79 (1.33–2.42); CM:2.06(1.40–3.03)	ACM: SA:1.49 (1.18–2.22), GNRI:1.02 (1.01–1.03), PNI:1.03 (1.01– 1.05); CM: GNRI:1.69 (1.19–2.44)	ACM,1.43(1.08-1.90)
Adjustment variables	Age, male, CR levels, SBP, history of CVD, history of DM, BUN levels, HB levels, use of ACEIs/ARBs	Age and sex	-	-	-	Age and sex	Nyha functional class, hypertension, DM, HF hospitalization, MI, stroke, AF, any cancer, use of ACEIs/ARBs, beta-blockers, HB levels, serum sodium levels, bilirubin levels, EGFR	Age, sex, HMI, systolic blood pressure, heart rate, prior HF, hypertension, cardiovascular disease, diabetes, and atrial fibrillation	-

ACM: all-cause mortality; CM: cardiovascular mortality; CEP: composite endpoint (all-cause mortality and HF rehospitalization); y: year; m: month; d: day.

### Meta-analysis result

#### SA

Three studies analyzed all-cause mortality with SA. After combining HR, lower SA predicted higher all-cause mortality in the random effects model (HR = 1.98; 95% CI = 1.282–3.057, p = 0.002; I^2^= 83.6%; Figure 2A ), and the Egger’s test (p = 0.584) did not identify publication bias. Three studies analyzed the composite endpoint of all-cause mortality and HF rehospitalization with SA, a fixed effects model (HR = 1.768; 95% CI = 1.483–2.108, p = 0.000; I^2^= 22.3%; Fig 2B ) was statistically significant, and Egger’s test (p = 0.661) showed no publication bias.

**Figure 2:** – A) Forrest plot of hazard ratio (HR) for the association between serum albumin (SA) and all-cause mortality; B) Forrest plot of the hazard ratio for the association between SA and the composite endpoint of all-cause mortality and HF rehospitalization; C) Forrest plot of the hazard ratio for the association between geriatric nutritional risk index (GNRI) and all-cause mortality; D) Forrest plot of the hazard ratio for the association between GNRI and cardiovascular mortality; E) Forrest plot of the hazard ratio for the association between prognostic nutritional index (PNI) and all-cause mortality. Heterogeneity among studies was determined using I2 statistics at a significance level of p < 0.05. CI: confidence interval; HR: hazard ratio.

#### GNRI

Four studies analyzed all-cause mortality with GNRI. After combining HR, the lower GNRI and the worse all-cause mortality were predicted. Since a significant heterogeneity was observed between individual studies (I^2^= 90.4%, p < 0.01), a random effects model was used to obtain the pooled estimate effect. The meta-analysis revealed a significantly increased all-cause mortality (HR: 1.812; 95% CI: 1.064–3.086, p = 0.029; Figure 2C ) for HFpEF patients with lower GNRI. However, there may be publication bias, as supported by Egger’s test (p = 0.014). This was tested further by Trim and Fill analysis, and the result of pooled HR did not change. The bias did not affect the evaluation result.

Cardiovascular mortality was analyzed in three studies with GNRI. Comprehensive data showed that lower GNRIs were related to higher cardiovascular mortality, and the fixed effects model (HR = 1.922; 95% CI = 1.504–2.457, p = 0.000; I^2^= 0.00%; Figure 2D ) was statistically significant, and Egger’s test (p = 0.41) showed there was no publication bias.

#### PNI

PNI was estimated using a random model in two studies, and the pooled HR revealed no statistical difference in all-cause mortality between the patients with a high and low level of PNI (HR: 1.176; 95% CI: 0.858–1.612, p = 0.314, I^2^=80.6%; Figure 2E ), and Egger’s test (p < 0.05) showed certain publication bias, as seen in some studies.

#### Study quality

The quality of these studies was assessed according to the Quality of Prognosis Studies Tool; seven studies ranked moderate quality, and two studies were ranked high quality. Four studies did not record or control confounding factors related to the evaluation results, and three did not provide information on losses to follow-up. Details are provided in Table 2 .

**Table 2:** – The Quality of Prognosis Studies Tool for assessing the quality of selected studies

Study, year	Study participation	Study attrition	Prognostic factor measurement	Outcome measurement	Study confounding	Statistical analysis and reporting	Total
MingLiu, 2012	M	L	M	M	H	M	M
Yoshiharu, 2013	M	L	M	M	L	M	M
Yu-Lu, 2017	M	M	M	M	L	M	M
Vasiliki, 2018	M	L	M	M	M	M	M
Toshiyuki, 2018	M	M	M	M	L	M	M
Isao, 2019	M	M	H	H	H	H	H
Shih Chieh, 2019	M	M	H	H	H	H	H
Masatoshi, 2019	M	M	M	M	M	M	M
Stuart, 2020	M	M	H	H	L	M	M

L: low quality; M: middle quality; H: high quality.

## Discussion

Malnutrition may result in energy deficiency, immunologic hypofunction, and tissue and organ damage.^32^ Compared with well-nourished patients, malnourished patients have longer hospital stays, higher readmission rates, and mortality.^33^ The imbalances of anabolism and catabolism in the development of HF can also lead to malnutrition. Research suggests that 50% of patients with chronic HF developed some degree of malnutrition.^25^ The imbalance between nutrient supply and energy needs results in impaired cellular energy metabolism and impacts the whole body’s metabolic systems. Significant body energy consumption can cause cardiac cachexia; it has been reported that 15% of patients with HF manifested cachexia.^25^ At the same time, cardiac cachexia is considered a risk factor for mortality in patients with HF.^34^

As the significance of malnutrition in patients with HFpEF has not yet been fully assessed, we evaluated the role of different nutritional indicators (SA, GNRI, and PNI) in predicting the disease prognosis in patients with HFpEF. We found that lower SA and GNRI scores were significantly associated with higher all-cause mortality, and lower SA is also associated with increased composite outcomes of all-cause mortality and HF hospitalization rates. However, there was no correlation between lower PNI and all-cause mortality. The findings suggest that SA and GNRI may be helpful indicators for prognosis assessment in a patient with HFpEF.

SA is a simple and objective indicator of nutritional evaluation, and it can better reflect muscle mass and protein storage.^35 , 36^ It is also considered an inflammatory marker.^37^ SA plays an important role in many physiological processes, including maintaining a stable colloid osmotic pressure and microvascular integrity, delivering substance in the body as a carrier protein, and scavenging free radicals and anticoagulant activities.^38^ According to a survey by Liu et al.,^20^ SA deficiency was observed in 30% of patients with chronic HF.^20^ Hypoproteinemia can promote the development of HF by causing pulmonary and myocardial edema, fluid retention, diuretic resistance, oxidative stress, and inflammation.^39^ A multicenter study including adults without HF has shown the important role of SA in the development of HF, in which baseline hypoalbuminemia is associated with an increased risk of developing HF during the 10-year follow-up period.^40^ However, studies have yielded conflicting results on the ability of SA to predict the prognosis of patients with HFpEF. Liu et al.^20^ suggest that hypoalbuminemia was significantly related to the increased risk of death for patients with HFpEF.^20^ However, Shanmugam et al. show that hypoalbuminemia had no obvious relationship to 1-year mortality in patients with HFpEF.^21^ Our meta-analysis reveals that hypoalbuminemia was significantly associated with a high all-cause mortality rate and HF hospitalizations in patients with HFpEF, which support that SA is a strong predictor of adverse outcome in patients with HFpEF.

GNRI was proposed by Bouillanne et al.,^41^ and its basic parameters are SA and body mass index (BMI). It was initially used to assess nutritional risk in the elderly. However, it was also found to predict clinical outcomes under different pathological conditions.^42 , 43^ Seoudy et al.^44^ suggest that compared to healthy individuals, the level of cardiovascular biomarkers increased markedly, and the prevalence of chronic HF was higher in patients with low GNRI.^44^ In addition, research showed that GNRI was associated with volume overload,^45^ higher cardiovascular death, and higher rates of rehospitalization^46 , 47^ in patients with HF. Our meta-analysis also indicates that low GNRI correlates with a high cardiovascular mortality rate in patients with HFpEF. Some researchers believe GNRI represents the patients’ frail state caused by various stressors under multiple systems disorders.^48 , 49^ Studies have shown that HFpEF patients have a higher mortality rate when they have low BMI and poor protein reserve,^35 , 50^ and this poor nutritional status may represent the progression of HFpEF.

PNI is a synthetically nutritional evaluation index representing protein synthesis and the body’s immune function.^51^ Nutritional state may affect the metabolism and function of immune cells, and malnutrition can lead to immunosuppression and affects prognosis in patients.^52^ PNI was originally used to assess the perioperative risk of gastrointestinal surgery patients.^53^ However, recent research shows that PNI is an effective prognostic marker in patients with various malignant tumors,^54^ acute HF,^25^ and pulmonary embolism.^55^ In our analysis, only two studies could be used for the combined analysis of the impact of low PNI on all-cause mortality of HFpEF, and the results failed to show a correlation between PNI and HFpEF. This lack of correlation may be due to clinical heterogeneity, as the cut-off points for PNI are not uniform. However, due to the small number of included studies and the unavailability of further subgroup analysis, high-quality studies are needed to evaluate the predictive value of PNI on the prognosis of HFpEF.

As HFpEF is a disease with high heterogeneity and complicated pathological processes caused by multiple comorbidities that can affect the development of HFpEF, a single nutritional index may not accurately predict the outcome in all patients. Comprehensive assessment of various nutrition indicators can provide complete prognostic information, and it would increase the ability to predict and risk stratification of HFpEF. At the same time, such risk identification may lead to improved clinical decision-making to delay disease progression, and formulating nutritional intervention plans may also help improve the clinical outcome of such patients. It has been shown that nutritional supplements are good for patients with chronic HF,^56 , 57^ but further clinical studies are needed to verify whether it is directly related to the prognosis of patients with HFpEF.

### Limitations

There are some limitations in our study. There are relatively few related studies; therefore, we could not include as many assessable studies as possible. In our meta-analysis, we defined HFpEF as an LVEF≥40%, which can cause a difference to some extent in the results. In some studies, the HR and 95% CIs were estimated by Kaplan–Meier survival curves, which may lead to potential error. Moreover, studies have a certain heterogeneity, which may be associated with the inconsistency of cut-off value and adjusted confounding factors when calculating HR in the included studies. In addition, as systemic diseases can affect nutritional status, this will also increase the heterogeneity of the study. Because of the limited number and quality of the studies, further studies are needed to evaluate the role of nutritional indicators in predicting the prognosis of HFpEF.

## Conclusion

As summarized in the central illustration , this meta-analysis provides evidence of the correlation between the nutritional indices, SA and GNRI, and the prognosis of HFpEF patients, showing that HFpEF patients with low SA have a higher risk of all-cause death and a higher risk of composite endpoint events of all-cause death and rehospitalization, and HFpEF patients with low GNRI have a higher risk of all-cause death and cardiovascular death. These results indicate the predictive value of SA and GNRI in the prognosis of HFpEF patients, and they may be useful reference indicators for the prognosis evaluation of HFpEF.

**Central Illustration:** : Prognostic Significance of Nutrition-Associated Markers in Heart Failure with Preserved Ejection Fraction: A Systematic Review and Meta-Analysis
